# Visiting mine reclamation: How field experience shapes perceptions of mining

**DOI:** 10.1007/s13280-024-02055-y

**Published:** 2024-07-29

**Authors:** Kamila Svobodova, Vojtěch Barták, Markéta Hendrychová

**Affiliations:** 1 Department of Agricultural Economics and Rural Development, University of Göttingen, Platz der Göttinger Sieben 5, 37073 Göttingen, Germany; 2 Faculty of Environmental Sciences, Czech University of Life Sciences Prague, Kamýcka 1176, 16521 Prague 6, Czech Republic; 3 Vršovická 663/73, 100 00 Prague 10, Czech Republic

**Keywords:** Attitude, Czech Republic, Extractive industry, Mine rehabilitation, Social–ecological interactions, Students

## Abstract

Recognizing the prevailing negative public opinion on mining, it is important to understand how firsthand encounters with mining activities might influence these perceptions. This study investigates how field trips to open pit coal mines and their reclamation sites in the Czech Republic affected the attitudes of 148 university students toward mining and mine reclamation. Using pre and post trip questionnaires, we observed significant changes: Students became less neutral about mining, saw it as a temporary disruptive activity, expressed reduced concern for social conflicts in mining areas, and showed increased support for the ecological restoration of post mining sites. These findings underscore the transformative impact of direct engagement with mine reclamation activities on shaping attitudes. Understanding these effects offers promise for positively shifting public perceptions of mining practices, emphasizing the potential for constructive changes in attitudes through field experiences with reclamation efforts in the Global North.

## Introduction

Almost every mineral and metal utilized today originates from mining, and the demand is on the rise. As governments need to adjust to emerging energy, environmental, and technology developments, various commodities will come in and out of fashion with the market. This period of transition will result in many mining projects opening and many mines closing, as shown by Svobodova et al. (2022).

Open-cut mining is one of the most serious environmental altering activities of human history, producing largely irreversible changes to landscapes (Hendrychová et al. 2020; Aska et al. 2023). No other industry has been able to significantly reshape the Earth's surface on such a vast scale, leading to enduring modifications that often outlast generations. The magnitude of social–ecological disruption caused by open-cut mining remains unparalleled, marking it as a foremost contributor to long-term environmental transformations and challenging the sustainability of affected ecosystems for decades, if not centuries, to come (Worlanyo and Jiangfeng 2021).

In the past, the mineral markets' volatility and the industry's recurrent boom-and-bust cycles often led to abrupt closures, leaving sites entirely abandoned by insolvent companies. This resulted in numerous abandoned and inadequately remediated mine sites, eventually becoming the responsibility of national or regional governments (Unger 2017). However, since the 1990s, there has been a growing acknowledgment within best-practice guidelines of the crucial role that closure and remediation play in both the ecological and economic sustainability of individual mines and the industry (Omotehinse and De Tomi 2023; Worden et al. 2024). Regulations governing mine closure have also become more robust (Syahrir et al. 2021). Nevertheless, closure planning continues to receive less attention compared to the development and operational phases and remains largely focused on the physical aspects of remediation while neglecting the impacts on the complex social–ecological systems (Bainton and Holcombe 2018; Arratia-Solar et al. 2022; Yu et al. 2024). Bridging this gap necessitates a holistic approach that integrates physical restoration and comprehensive considerations of social–ecological systems, particularly the diverse interactions between people and mines.

Direct engagement with mining sites and the observation of reclamation practices present a valuable education on the multifaceted impacts of mineral extraction (Svobodova 2023). These experiences can provide tangible insights into the complexities and challenges inherent in extraction while emphasizing the critical need for sustainable practices. Observing and learning about reclamation projects, where mined land is restored and rehabilitated, can showcase the government's and industry's dedication to environmental responsibility and align with the broader goal of enhancing community awareness and participation in decision-making processes related to mining activities. This direct exposure can serve as a catalyst for a more comprehensive understanding of the necessity for socially and environmentally aware strategies in both the mining sector's operations and the formulation of national regulatory frameworks.

While numerous theoretical guidelines exist to enhance the mining industry's image and public perception (e.g., ICMM 2019), none of these tools is grounded in hands-on opportunities such as field trips. A positive attitude toward mining holds immense significance for industry, government, and society as it forms the bedrock for sustainable collaboration and progress (Svobodova et al. 2020). A positive perception fosters community engagement, reduces conflicts, and establishes a common ground for dialog, enabling mining activities to align better with community needs and expectations.

Our paper investigates the effect of field trips to mining and reclamation sites on the attitudes held by university students. While conducted on a student sample, this pioneering research offers insights that can serve as a cornerstone for future investigations in this domain. It possesses the potential to provide guidance and direction for further research endeavors, shaping a more comprehensive understanding of the influence of such experiences on attitudes within broader populations.

## Social–ecological *nexus* surrounding mining

The social–ecological nexus surrounding mining represents a complex interplay between mining activities and their impacts on social systems and ecological environments. On the one hand, mining can provide economic opportunities, jobs, and infrastructure development for communities, particularly in resource-rich regions. However, these benefits are often accompanied by significant social and environmental costs. Communities located near mining sites often face social challenges, ranging from socio-economic disparities to cultural disruptions, resettlements, and health concerns (e.g., Schueler et al. 2011). The influx of mining operations can lead to demographic shifts, putting pressure on local infrastructure and services. This can strain social cohesion and exacerbate tensions between newcomers and long-standing residents, particularly in cases where indigenous or marginalized communities are affected (see Bainton 2020).

Le Gouill and Poupeau (2020) introduced a framework to evaluate mining production as a social–ecological system, offering a nuanced view of extractive activities as “systems of interdependent resources.” Their work underscores the territorial dimension inherent in extraction processes. This system-oriented approach to mining resonates with the research of Nilsson et al. (2021), who developed an analytical framework of the socio-ecological–technological impacts of mining in the Nordic Arctic.

Mine rehabilitation is integral to the social–ecological nexus. Well-rehabilitated mine sites can provide habitat for wildlife, promote soil health, and contribute to essential ecosystem services such as water purification and carbon sequestration (Hendrychová et al. 2020). This restoration not only benefits local ecosystems but also enhances the well-being of nearby communities by preserving natural resources and ecosystem functions essential for their livelihoods. It is pertinent to acknowledge that while ecosystem services are interconnected with the social–ecological dynamics of mining, as demonstrated by studies such as Boldy et al. (2021) and Larondelle and Haase (2012), this paper focuses specifically on attitudes toward mining and mine reclamation, along with their policy implications, rather than delving into the broader concept of ecosystem services.

### Attitudes toward mining

Attitudes toward mining and mine reclamation play a crucial role in shaping the social–ecological nexus surrounding mining. These attitudes can influence public perception, policy decisions, and stakeholder interactions, shaping the trajectory of mining activities and their impacts on both people and the environment.

Attitudes toward the environment are complex and based on cognitive beliefs about the world and its ecology (Larson 2010). These beliefs strongly support human evaluative attitudes and opinions that are mostly changed only with deeper transformation in socio-demographic characteristics or with direct experience (Heberlein 2012). Attitudes represent people’s general evaluations of humans, objects, and issues (Petty and Briñol 2015). However, they are not stable concepts and can be changed under the influence of various aspects. Understanding this, researchers have developed numerous theories to account for the psychological process underlying attitude change and to identify variables that can affect this change (e.g., Kruglanski and Thompson 1999; Briñol and Petty 2012; Chaiken and Ledgerwood 2012; Kruglanski 2012). For example, Petty and Briñol (2015) demonstrated that attitudes can be changed through the operation of source, message, recipient, and context factors affecting both low and high thought processes. Wood (2000) assumed that informational and normative motives are each associated with unique mechanisms that generate unique forms of attitude change. Socio-demographic characteristics, sources of information, and social networks can also affect attitude change as demonstrated by Heberlein (2012) and Zimbardo (1991). JRank (2016) substantiated a direct correlation between attitude shifts and self-esteem, highlighting the significance of communication methods in this process. Particularly, face-to-face communication was found to be notably more effective than mass communication channels in fostering such changes.

Direct experience can significantly influence attitude change as shown by Nyrstrand and Fjørtoft (2015), Jensen et al. (2013), and Nairn (2005). In this way, Hope (2009) argues that direct experience can deepen and develop the understanding of the current knowledge gained by previous experiences and education. According to Hope, direct experience provides the opportunity to pursue our current understanding shaped by many aspects and opinions further in a particular real-world context. Considering deeper effects, attitude change can mediate the impact on behavior change (Fishbein and Ajzen 1975; Huijts et al. 2012; Sovacool 2014).

### The aim of the study

This article aims to analyze the effect of direct experience with mining and mine reclamation on the attitudes toward mining. We examine whether and to what extent attitudes toward mining can be changed after direct field experience, and how this change is influenced by the characteristics of participants.

Our study aligns with the approach outlined by Krosnick and Smith (1994), defining direct experience as direct contact with an object or personal experience involving the object. Building upon this approach, we followed the methodology proposed by Duerden and Witt (2010) by orchestrating direct experiences with mining activities through a field trip involving student participants. By investigating the shifts in students’ attitudes throughout this excursion to mining and reclamation sites, we aim to uncover valuable insights into the dynamic nature of attitudes toward mining. Understanding how these experiences shape the perceptions and beliefs of participants could shed light on the interplay between direct encounters and attitude formation, offering nuanced insights into the efficacy of firsthand experiences in altering attitudes toward mining and mine reclamation.

## Materials and methods

### Geographical and regulatory context of the study area

**Fig. 1:**
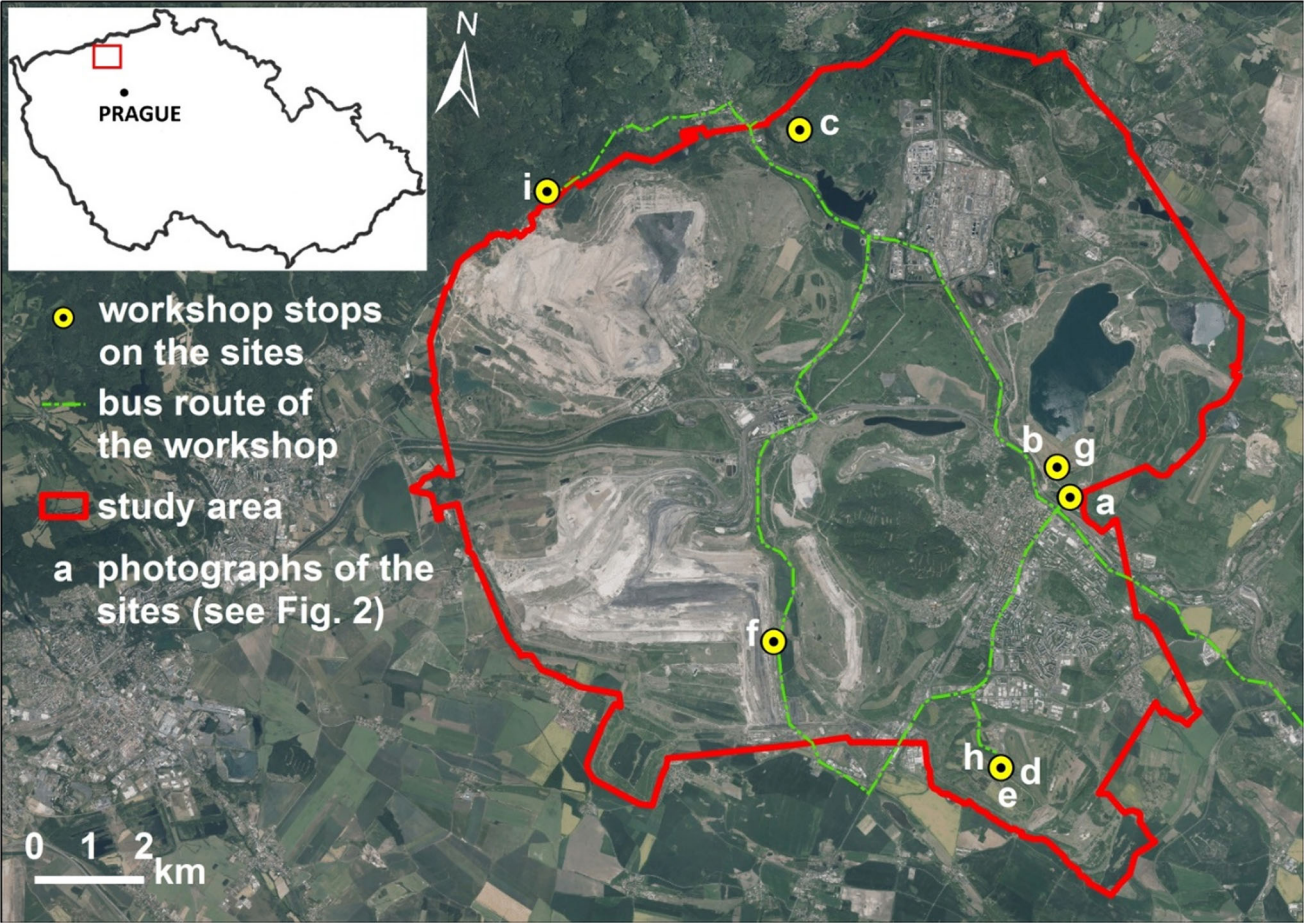
The study area of the Most lignite basin is located in the north-west of the Czech Republic. The study area includes two open-pit mines, Jan Sverma and CSA, depicted on the left side of the study area, and several rehabilitated sites, such as Cepirozska, Slatinicka, Velebudicka dump, or the Most lake. The yellow spots present localities shown in Fig. 2

The study area lies within the Most lignite basin in the north-western region of the Czech Republic (see Fig. 1). Open-cut mining activities have significantly impacted the Czech Republic, ranking it among the most mine-affected European countries. A total of 135 villages and towns have vanished due to coal mining, with 62 of these settlements situated within the lignite basin (Yilmaz and Marschalko 2012). Presently, five open-pit mines are actively conducting surface lignite mining operations. The aftermath of extensive mining led to the initiation of large-scale post-mining reclamation practices from around the 1950s onwards. A substantial portion of the previously mined land has undergone mine reclamation, rendering it accessible to the public (Hendrychová and Kabrna 2016).

The legal framework governing mining activities in the study area, and throughout the Czech Republic, is established by the National Mining Act from 1988 (Czech Republic 1988). According to this act, mining companies are legally obligated to rehabilitate all areas affected by mining activities at their own expense. To ensure the quality of mined land rehabilitation, companies pay a fee for every ton of excavated coal into a state-owned bank account overseen by the National Mining Council. This fee is calculated as part of the licensing application process and is based on the size of the resource and an estimation of the extent of planned rehabilitation works. The National Mining Council conducts annual on-site reviews to assess the success of rehabilitation efforts and release the budget for the following year accordingly. Although there was an update to this legislation in 2021, the procedure remains unchanged, as does the mine rehabilitation practice, which is also subject to other national legislation such as forestry, agricultural, and environmental impact assessment regulations.

### Data collection and the sample structure

**Fig. 2:**
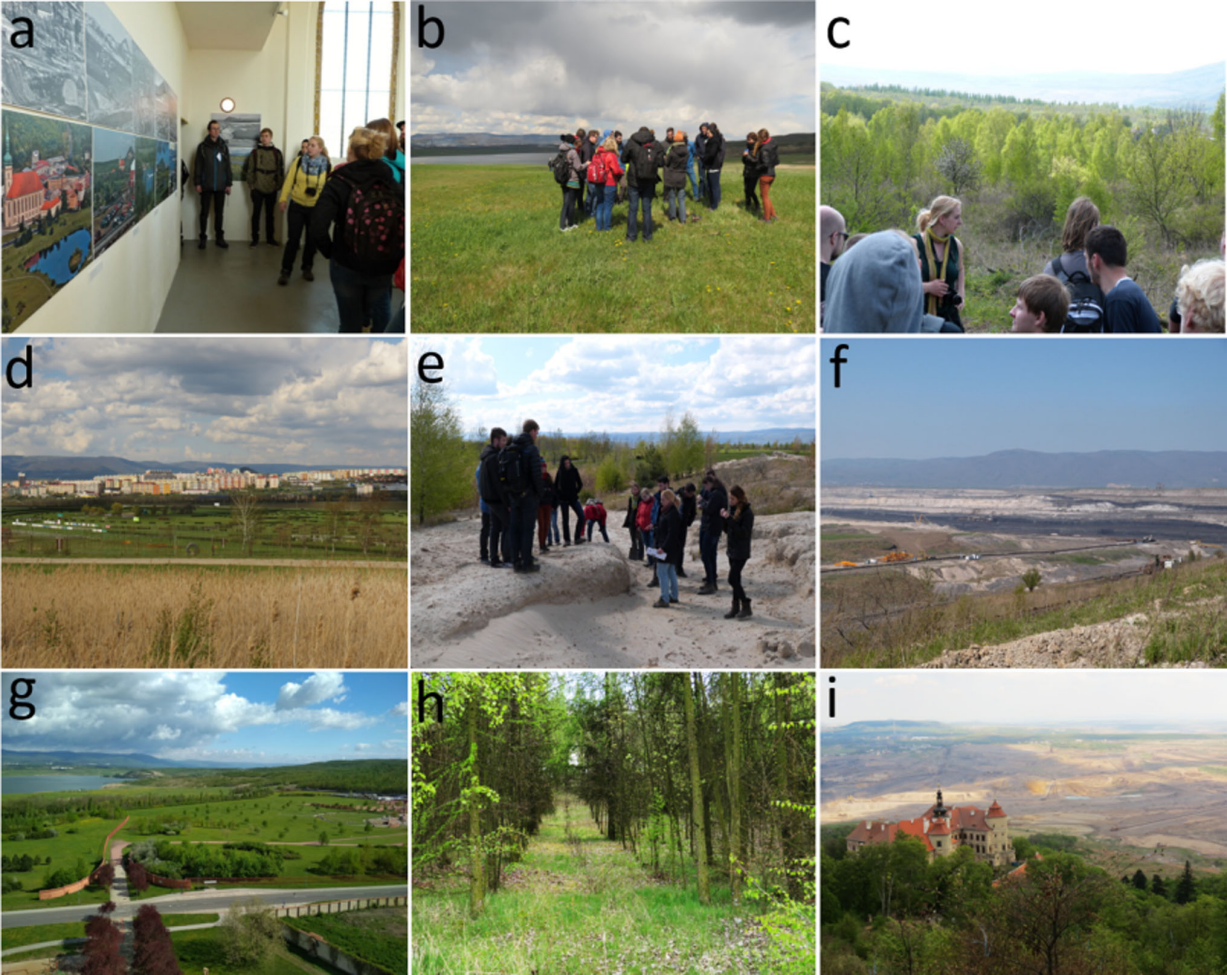
The field trip program showcased the on-site photographs: **a** the photo exhibition on mining excavation and mine reclamation in the study area; **b** the forest reclamation of the site Lezaky according to a reclamation plan; **c** the naturally restored post-mining site including a mixed forest with open patches combined with small wetlands—30 years of ecological succession; **d** the Most Hippodrome as a community aimed reclamation in the urbanized area; **e** the ecologically restored sandy post-mining site with rare aculean species—10 years of ecological succession; and **f** the open-pit CSA as seen from a viewpoint

Five field trips that were organized in May 2016 (three trips) and in May 2017 (two trips) in the study area of the Most lignite basin. Each field trip took 7 h. The weather was sunny and warm during the field trips. The program of the trips was planned with the same timing and schedule for all five trips. First, students saw a photo exhibition about landscape changes in the study area due to mining excavation and mine reclamation. The photographs were taken in the period from the 1970s until the present and showed the history of mining and mine reclamation activities in the study area. Second, participants visited the reclamation site Lezaky restored according to a reclamation plan to water areas, forests, and agricultural land. Third, participants visited two post-mining sites restored naturally using ecological succession principles. The sites host natural habitats such as sandy sites with rare aculean species and mixed forests with open patches combined with small wetlands in different phases of ecological succession (30 years old and 10 years old ecological restoration). Fourth, they visited the Most Hippodrome which represents a community-focused reclamation. At the end of the field trip, students visited two viewpoints at the edge of an open-pit Jan Sverma and a pit CSA providing views on coal excavation of large scales (see Fig. 2).

A total of 148 students took part in the field trips and the survey. All students were informed about this research before the trip. Their characteristics are listed in Table 1. The selection of a homogeneous sample of student respondents was guided by Zube (1974) who confirmed low differences between the responses of different groups of respondents. According to the program, the participants were bussed to the study area where they spent 7 h at various locations. All participants were Master's students at the Czech University of Life Sciences Prague aged between 22 and 28 years. All of them had completed the course Land Reclamation and successfully passed the examination 5 months before the field trip; therefore, the field trip was not a condition for the examination. However, the participants had assumable higher knowledge of the theory of mining and mine rehabilitation, almost 80% of them had no previous experience with reclamation beyond the university course. In addition, over 30% of them had never visited a mine reclamation site, and over 26% had never visited a mine.

**Table 1:** Participants’ characteristics and corresponding predictors used in the cumulative logit models in data analyses

Variable	Question	Characteristics, % of the sample	Predictor
Gender	What is your gender?	Man 49.3% Woman 50.7%	P1
Environmental attitude	In what environmental attitude theory do you believe?	Ecocentrism 89.2%; Anthropocentrism 10.8%	P2
Previous visit to a surface mine	Have you visited a surface mine before this trip?	Frequent (More than two) 35.8% Rare (One or two) 37.8% No 26.4%	P3
Previous visit to a reclaimed site	Have you visited a reclaimed site before this trip?	Frequent (More than two) 33.1% Rare (One or two) 35.8% No 31.1%	P4
Previous experience beyond the trip	Do you have another previous experience with mining and mine rehabilitation beyond your study?	Yes 20.9% No 79.1%	P5
Source of information about mining	Please, identify the main sources that you use to obtain information about mining	*Study, work:* Yes 85.8% No 14.2%	P6
		*Internet:* Yes 29.1% No 70.9%	P7
		*Other media (TV, radio, print newspapers, and journals):* Yes 16.2% No 83.8%	P8

A reclamation planner working for the mining company operating in the study area guided the field trips. Participants had no previous experience with this professional that avoided undesirable influence on participants in terms of their relationship with a guide (Petty and Briñol 2015). During the field trips, the guide did not talk about the topics of the statements used to analyze the students’ attitudes in this research.

Two paper questionnaires were developed to collect the data. One questionnaire was used before the field trip (pre-w) and the second one after the trip (post-w). The pre-w questionnaire was divided into two parts. The first part contained questions on the participant’s name, environmental attitudes, previous visit to a surface mine, previous visit to a reclamation area, previous experience with mine reclamation beyond the course, and their main source of information about mining. The second part of the pre-w questionnaire was designed to measure participant’s attitudes toward mining. It contained eight attitudinal statements divided into three groups focused on different aspects of mining and mine reclamation as shown in Table 2. Participants were asked to evaluate each of these statements on the 5-point Likert scale according to their agreement with the statement: strongly disagree (1), disagree (2), neither agree nor disagree (3), agree (4), and strongly agree (5)—as guided by Bailey (1987). The post-w questionnaire contained only a question on the participant’s name and the identical statement part as in the pre-w questionnaire. The two-step questioning was designed to measure the attitude change due to the direct experience in the field trips. Participants completed both questionnaires independently while bussing to and from the trip, i.e., shortly before and after the experience. They spent around 12 min completing the pre-w questionnaire and about 8 min completing the post-w questionnaire.

**Table 2:** Statements used in the questionnaire were sorted according to the aspect of mining and mine reclamation

Aspects of mining/groups of statements	Statement	
Environmental impacts of mining	S1	Destructive environmental impacts of mining operations are temporary
	S2	A post-mining reclamation site cannot achieve such environmental quality as it had before mining
Social and economic impacts in the region	S3	Mining operations have primarily a positive economic impact on local communities
	S4	Mining operations increase local social conflicts
	S7	Mining is an opportunity to create a landscape that will be unique and valuable for the region
Procedures and planning in mine reclamation	S5	Ecological restoration without human assistance (ecological succession) should be a part of each post-mining area, regardless of its high time demands
	S6	Local communities connect better with the reclaimed land if they can participate in its design from the beginning
	S8	The regulatory and legislative systems in the Czech Republic related to mining activities are adequate and sufficient

### Data analysis

The responses to the eight statements were coded by numbers from 1 (“strong disagreement”) to 5 (“strong agreement”), quantifying the attitudes of participants. The change in attitudes was calculated as the attitude after the field trip minus the attitude before it. For each question, we tested the hypothesis of zero change in attitude using the Wilcoxon test. To assess the possible relation between the attitude before the excursion and the characteristics of the respondents, we fitted an ordinal cumulative logit model for each question separately, with gender (P1), environmental attitude (P2), previous visits to a surface mine (P3), previous visits to a reclaimed site (P4), prior experience with mining and/or mine rehabilitation beyond the university field trip (P5), and source of information about mining (P6–P8) as categorical predictors. The same approach was also applied to assess the relationship between students’ characteristics and the change in their attitudes. For all analyses and data manipulations, we used the R statistical software (R Development Core Team 2008) together with the package “ordinal” (Christensen 2015).

To evaluate the significance of each predictor, we assessed its marginal effect by comparing the null model (with no predictors) with the model only with the predictor under evaluation (“add1” function in R). We used this approach as we expected strong dependencies among the predictors, which could lead to multi-collinearity problems if we used them together in one model. Thus, our models provide a rather descriptive evaluation of each predictor while not controlling for the effects of other predictors. All model comparisons were realized as likelihood ratio tests. For models with at least a weakly significant marginal effect (i.e., *p* < 0.1), we then assessed the significance of the odds ratio for each pair of levels of each predictor by Wald tests and Wald confidence intervals. The odds ratios were used to compare a “tendency to agree” with the attitudinal statements resp. a “tendency to change the attitude toward agreement” between the predictor levels. Since all our predictors had no more than three levels (see Table 1), we did not apply any corrections for multiple comparisons.

## Results

**Fig. 3:**
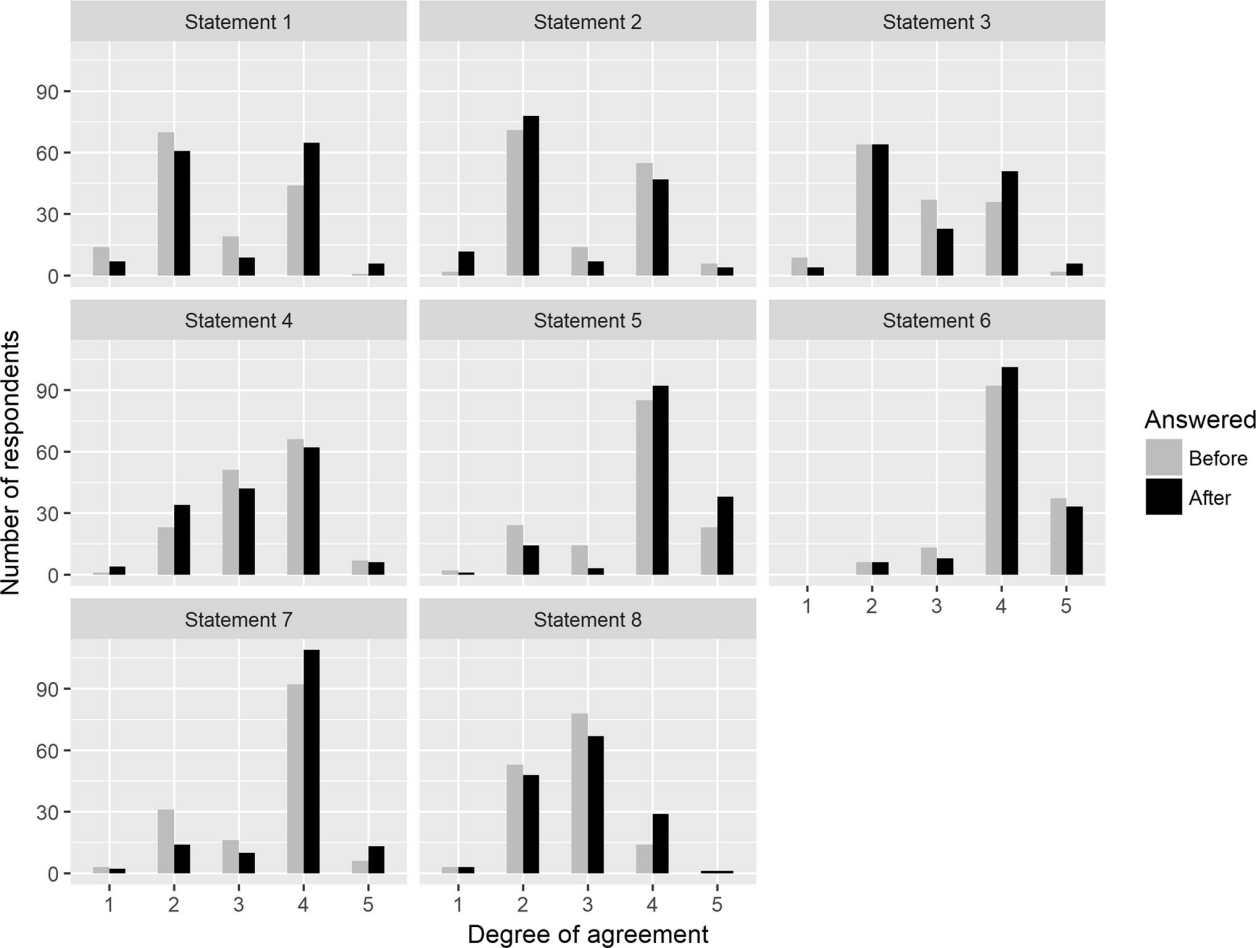
The distribution of participants' responses to all eight attitudinal statements before and after the field trip

**Fig. 4:**
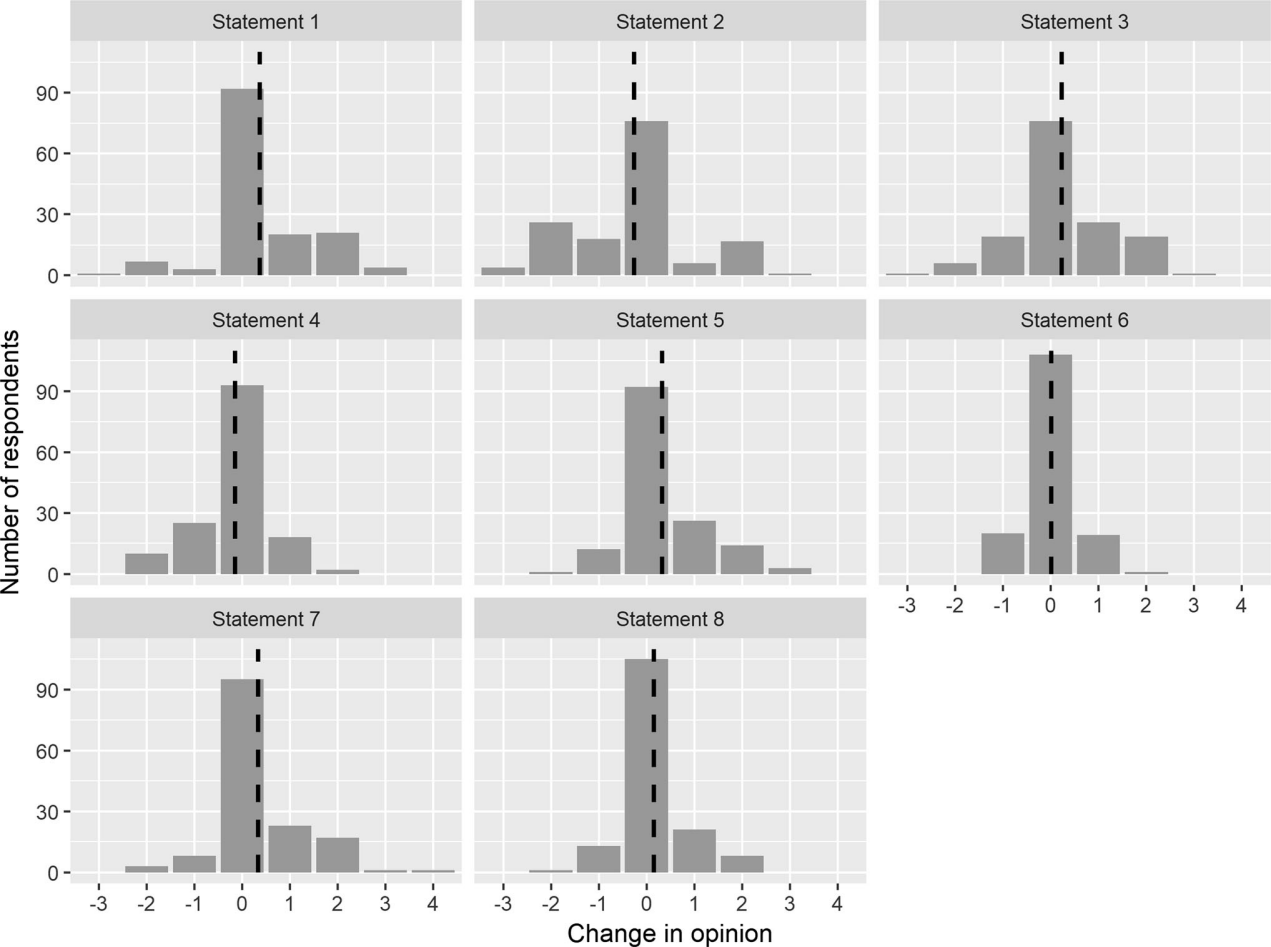
The histogram showcases the distribution of attitude changes across all eight statements. The thick dashed line indicates the mean change, serving as a central reference point. Zero signifies no change, positive values indicate a shift toward a greater agreement with a statement, and negative values signify a move toward greater disagreement with a statement

The distribution of answers before and after the field trip is shown in Fig. 3. Wilcoxon tests revealed significant changes in opinions across all statements, barring statement S6 (Table 2). These alterations generally leaned toward either agreement or disagreement, with the highest absolute mean change recorded at 0.36 (as detailed in Table 3 and Fig. 4). Moreover, the findings indicate a notable decrease in the frequency of neutral responses after the field trip compared to before. This shift was consistent across all statements (as illustrated in Fig. 3), showcasing a mean (± SD) frequency decrease of − 9.1 (± 3.0).

**Table 3:** Mean and standard deviations of attitude changes for each statement. The associated p-values correspond to the Wilcoxon tests examining nonzero changes in attitudes. A negative mean change indicates a decline in agreement, whereas a positive mean change signifies an increase in participants' alignment with the statement

Statement	Mean change	SD	*p* value
(S1) Destructive environmental impacts of mining operations are temporary	0.36	1.041	0.0002***
(S2) A rehabilitated area cannot achieve such quality as it had before mining	− 0.26	1.249	0.0252*
(S3) Mining operations have primarily a positive economic impact on local communities	0.22	1.025	0.0078***
(S4) Mining operations increase local social conflicts	− 0.16	0.769	0.0143*
(S5) Ecological restoration without human assistance (ecological succession) should be a part of each post-mining area, regardless of its high time demands	0.33	0.857	0.0000***
(S6) Local communities connect better with the rehabilitated land if they can participate in its design from the beginning	0.01	0.539	0.8848
(S7) Mining is an opportunity to create a unique and valuable landscape after the mine rehabilitation	0.34	0.897	0.0000***
(S8) The regulatory and legislative systems related to mining activities are adequate and sufficient	0.15	0.671	0.0080***

The most notable change in participants’ attitudes was found with the statements S1, S5, and S7 (see Table 3 and Fig. 4). Specifically, 36% of participants changed their attitudes toward mining regarding mining's potential to create a unique and valuable landscape (S7). Before the field trip, 66% of participants agreed that rehabilitated mining land could transform into a valuable landscape, with 23% in disagreement and 11% neutral. Post-field trip, this agreement surged to 82%, accompanied by a decrease in disagreement to 11%, and 7% maintaining a neutral stance. Similarly, 38% of participants changed their attitudes toward ecological restoration (S5). Initially, 73% supported ecological restoration via succession in post-mining areas, irrespective of its time-intensive nature, while 18% disagreed, and 9% were neutral. Following the field trip, support for ecological restoration amplified to 88%, with opposition decreasing to 15%, and only 3% remaining neutral. Regarding the perception of mining's destructive environmental impacts (S1), 38% of participants altered their viewpoints. Pre-field trips, only 30% believed these impacts to be temporary, while 57% disagreed, and 13% were neutral. Post-field trip, 48% perceived these impacts as temporary, with 46% still against this notion, and 6% maintaining neutrality.

In examining the influence of respondents' characteristics on attitude changes, intriguing patterns emerged. Participants who had not visited a surface mine showed a stronger inclination toward shifting their opinion to agree more strongly with the statement that a reclamation site could not match its previous environmental quality as it had before mining (S2), compared to those who had direct experience in such settings. Conversely, individuals with prior visits to reclamation sites displayed a higher propensity to shift toward stronger agreement with the statement that ecological restoration without human assistance should be a part of each post-mining area (S5) compared to those without such experiences. Moreover, the analysis revealed that attitude changes regarding the impact of mining operations on local social conflicts (S4) were marginally influenced by the participants' primary source of information about mining. Participants relying on academic studies as their primary information source were more prone to align their attitudes toward agreeing that mining escalates social conflicts. In contrast, those reliant on mass media tended an opposite shift in attitude, although this effect was relatively minor and carried marginal significance, as indicated by the corresponding odds ratios (see Table 4).

**Table 4:** The results of the cumulative logit model analysis, associating respondent characteristics with their attitude changes before and after the field trip. Interpretation of the values and significances is analogical to Table 1. The odds ratios can be interpreted as a tendency to change attitude toward agreement

Statement	Variable	Levels compared	Odds ratio	Confidence interval
**S2**	**Previous visit to a surface mine**	**No/Rare**	2.28*	**(1.04; 5.09)**
		No/Frequent	1.22	(0.54; 2.74)
		Frequent/Rare	1.87	(0.93; 3.81)
S4	Study = source of information about mining ⋅	Yes/No	2.23	(0.86; 5.68)
	Other media = source of information about mining	No/Yes	2.10	(0.88; 4.95)
**S5**	**Previous visit to a reclaimed site**	**Rare/No**	2.54*	**(1.12; 5.92)**
		Frequent/No	1.34	(0.57; 3.18)
		Rare/Frequent	1.89	(0.88; 4.09)

## Discussion

This study was designed to delve into the impact of field trips to mining and post-mining sites on individuals' attitudes toward mining. Engaging student participants, our research examined their attitudes both before and after a field trip to the Most region in the Czech Republic. Encouragingly, our findings indicated a discernible shift toward a more favorable view of mining following the field experience.

In summarizing our key findings, several insights have surfaced from our study regarding the impact of the field experience on attitudes toward mining. These noteworthy conclusions can be distilled into five key lessons:

1. Decrease in apathy toward mining: Participants exhibited a more pronounced shift in their attitudes toward mining following direct experience in mine and reclamation sites.
2. Temporal perception of mining: Direct exposure to mining and reclamation sites reinforced the perception of mining as a temporary, albeit destructive, activity.
3. Economic and social impacts of mining: Direct engagement with mine reclamation bolstered positive opinions regarding the economic benefits while alleviating concerns about social conflicts within mining regions.
4. Support for ecological restoration: Participants' support for the ecological restoration of post-mining sites notably increased after their direct experience with this form of mine recovery.
5. Impact of prior experience: Participants without prior exposure to surface mines demonstrated a higher inclination to align with the belief that a reclamation site could not fully regain its previous environmental quality compared to those who had direct experience in such settings.

### Decrease in apathy toward mining

Direct experience with mining and mine reclamation significantly reduced participants' neutrality or indifference toward the issue. Prior to the field trip, neutral responses accounted for 30.3% (± 23.6%) of answers, decreasing notably to 21.1% (± 22.4%) afterward. Furthermore, 10.7% (SD = 4.3%) of participants shifted their opinions from neutral to either positive or negative, while only 4.6% (SD = 3.1%) moved in the opposite direction, from positive or negative toward a neutral stance.

These findings echo studies by Dziewas (2008), Boyle et al. (2007), Paris and Turner (1994), and Kern and Carpenter (1986), emphasizing how direct experience amplifies interest, motivation, and understanding of the issue. Similarly, Hope (2009) and Fuller et al. (2006) noted that firsthand encounters significantly enhance participants' knowledge, shaping their opinions. Field visits, as described by Boyle et al. (2007), are perceived as a deeper form of learning, while Foskett (1999) highlighted a significant correlation between direct experience and cognitive enrichment. Aligning with our study, Riegel and Kindermann (2016) emphasized that firsthand experience fortifies perception and imparts meaning to the content.

### Temporal perception of mining

The most substantial shift in participants' attitudes post-field trip was observed in their increased acknowledgment of the destructive environmental impact of mining operations (see Fig. 4). While initially, 24.3% of participants perceived mining as inherently destructive, after visiting mining and reclamation sites, 15.5% altered their viewpoint, now seeing its negative environmental effects as temporary. Conversely, only 5.4% exhibited an opposite change in perception.

Our findings resonate with the concept emphasized by Petty and Briñol (2015) that the situation and context surrounding message presentation can profoundly impact the extent of attitude change. The field trips were meticulously designed to offer students in-depth insights and factual knowledge about mine reclamation, spanning a comprehensive 7-h exposure to post-mining environments. This immersive experience likely influenced participants toward a higher acceptance of the temporary destructive impacts of mining. This aligns with Dettmann-Easler and Pease's (1999) findings that outdoor contexts positively impact ecological attitudes. Furthermore, the sunny weather during these trips might have contributed to the observed shift toward more positive attitudes. Research by Keller et al. (2005) highlighted that exposure to sunlight significantly affects both mood and cognition. Additionally, seasonal effects, as discussed by Lambert et al. (2002), could have played a role. Spending time outdoors during spring, as explored by Keller et al. (2005), correlates with increased receptiveness to new information, improved mood, and cognitive abilities.

### Economic and social impacts of mining

Student participants exhibited notable shifts in their perspectives on the economic and social impacts of mining, particularly concerning the potential for creating a unique landscape after mining operations in the region. Before the workshop, 66.2% acknowledged this opportunity, a number that rose to 82.4% following the field trip. The change in their opinions correlates with their overall positive change of attitudes toward mining. Outdoor contexts, as discussed by Dettmann-Easler and Pease (1999), might have contributed to this change.

Regarding the positive economic impacts of mining on local communities, while the overall shift in attitudes among student participants was limited, 7.4% transitioned from a neutral stance to a positive one after the field trip. Before the field trip, 49.3% of students perceived increased social conflicts in mining regions linked to mining activities. Among those who initially agreed, a majority (37.2% of respondents) maintained their opinions. However, 13.5% shifted from a neutral or positive standpoint to disagreement, while 8.8% moved toward agreement. It is worth noting that while participants gained insights from the guide while passing affected municipalities by bus, they did not have direct interactions with local communities during the field trip.

### Support for ecological restoration

Before the field trip, a substantial 73% of participants already recognized ecological restoration as a vital component of the mine rehabilitation process. Almost all of them maintained this viewpoint post-excursion. However, the most notable shift in attitudes was observed among those initially holding neutral or negative opinions. Following the field experience, 29.1% of participants transitioned toward more positive attitudes regarding ecological restoration. This change could potentially be linked to the impact of nature experiences on fostering pro-environmental attitudes, a phenomenon highlighted by Dettmann-Easler and Pease (1999). The perception of wilderness, a characteristic feature of ecological restoration (Bradshaw 1997), might have influenced these shifting attitudes. Studies such as Curado et al. (2014) have demonstrated a greater preference for naturally restored landscapes over planned reclamation. However, research by Van den Berg et al. (1998) suggests that younger participants tend to express a higher preference for wilderness in nature compared to older individuals. Moreover, attitudes toward the ecological restoration of post-mining lands may also be associated with the educational background and focus of participants. Sklenicka and Molnarova (2010) and Van den Berg and Koole (2006) found that environmentalists and those with higher education exhibit more positive opinions about the wilderness in nature compared to others.

### Impact of prior experience

Participants with prior visits to surface mines and mine reclamation sites displayed more favorable opinions on mine reclamation and its environmental quality than those without such firsthand exposure. This aligns with research by Svobodova et al. (2020), illustrating that stakeholders directly acquainted with the mining sector hold notably more positive perceptions than other groups. Sonmez and Graefe (1998) reinforce this finding, highlighting past experience as a crucial determinant of future behavior.

### Study limitations and future research

We acknowledge certain limitations inherent in our study. Firstly, the data analyzed in this study were collected in 2016 and 2017. While not the most recent, they remain relevant and valuable, especially considering the limited attention this topic has received. However, we acknowledge that, since 2017, events such as global regulatory changes, technological advancements, economic fluctuations, significant mining-related incidents, and increased public pressure on climate change mitigation could influence the context of similar research conducted today. These factors are important to consider when interpreting our findings. Despite these potential impacts, the stable regulatory context and persistent challenges highlighted in our study ensure its continued significance. Most importantly, our results carry significant policy implications and highlight persistent trends and challenges that demand attention from policymakers, especially considering that the practice and regulatory context of mine reclamation in the study area have not undergone significant changes since the period of data collection, as described in section "Geographical and regulatory context of the study area."

Secondly, our research was conducted with a sample of student participants, aligning with the approach taken in attitudinal studies such as those by Svobodova et al. (2017), AlGhamdi et al. (2014), and Prabawa-Sear and Baudains (2011), which similarly focused exclusively on students. While it is recognized that the views of student participants may not fully represent those of the broader population, the homogeneity of the sample serves a purpose in illustrating variances among individuals and highlighting preferential trends, consistent with the observations of Zube (1974). We propose that delving into changes in students' attitudes before and after field trips offers valuable insights into the contentious issue of mining's impact on public opinion. However, future investigations should consider broadening the scope to encompass a sample more representative of the general public, eschewing reliance on a consistently homogeneous group of respondents. Additionally, international and inter-site comparisons merit attention.

Thirdly, the student participants possessed assumable higher knowledge of the theory of mining and mine reclamation practices as all of them had completed the course Land Reclamation 5 months before the field trip. The results of the same experiment may be different for a group of participants without such experience. Future research should adopt a more comprehensive approach by examining the mechanisms through which attitude changes occur. Factors such as participants' behaviors, emotions, feelings of fluency, and self-worth, as proposed by Petty and Briñol (2015) and JRank (2016), warrant closer scrutiny to better understand the extent of influence on attitude change.

The field trips were guided by a professional working in the study area, participants had no previous experience with the professional and, therefore, no personal relationship that could influence their perception. However, the personality of the guide may still exert influence, given findings by Petty and Briñol (2015) that highly credible individuals tend to wield more influence and induce greater attitude change. The credibility of a source, as emphasized by Petty et al. (1981), Chaiken and Maheswaran (1994), and Petty (1997), plays a pivotal role in shaping attitudes, with experts exerting a more substantial impact than non-experts. Thus, future research should consider the guide's character, program, and timeframe as variables, incorporating them into analyses due to their potential impact on participant attitudes.

Finally, this research study was conducted in the Czech Republic, a Global North country known for its relatively favorable natural conditions and stringent mining regulations. However, performing the same study in a Global South country might yield different results. Future research endeavors should adopt an international approach, encompassing case studies across diverse landscapes and legal frameworks.

## Conclusion

As we progress in the pursuit of sustainable mining practices, the emphasis on non-material assets, such as field experience and knowledge, becomes increasingly vital (Prno and Slocombe 2014; Svobodova et al. 2019). Our research underscores significant implications, particularly within the regulations of mining operations in the Global North. Despite the common tendency of governments to primarily address the adverse environmental impacts of mining operations in their regulatory practices, there is a noteworthy oversight regarding cognitive assets (Schiuma 2012; Lyytimäki and Peltonen 2016; Van der Plank et al. 2016). Enhanced knowledge derived from firsthand experience stands as a crucial intangible asset that warrants attention.

This pioneering research offers insights that can serve as a cornerstone for future investigations in this domain. The findings gleaned from this study possess the potential to provide guidance and direction for further research endeavors, shaping a more comprehensive understanding of the influence of such experiences on attitudes within broader populations.

We suggest that stakeholders’ direct field experience with mining and mine reclamation can help to better understand the complex social–ecological interactions within the area. This understanding is beneficial for both governments and the mining industry. By gaining firsthand experience of mining impacts and benefits, coupled with better communication from governments and mining companies, it is possible to foster social acceptance and understanding. This, in turn, could lead to better community relations, reduced conflicts, improved trust, and ultimately ensure that mining activities align with stakeholder needs and expectations.

By integrating the field experience of local stakeholders into governmental regulations, such as incorporating it into regular regulatory controls governing the mine rehabilitation process, governments can improve the effectiveness and sustainability of mining rehabilitation efforts. This approach can foster stakeholder engagement and ownership, ensuring that post-mining landscapes are restored in a manner that aligns with local needs and environmental considerations.

From the mining industry’s perspective, positive stakeholder field experiences may result in a favorable shift in stakeholder attitudes, benefiting the mining company. The positive attitudes can foster an environment where the industry can operate with support and collaboration from local stakeholders. Such an approach may facilitate smoother operations, enhance investor confidence and attract talent, ensuring the industry's sustainable growth. Moreover, by valuing landscapes beyond their economic contributions, governments and the mining industry can demonstrate respect for both the natural environment and the diverse human experiences intertwined with it.
